# Feed competition reduces heritable variation for body weight in *Litopenaeus vannamei*

**DOI:** 10.1186/s12711-020-00565-3

**Published:** 2020-08-08

**Authors:** Sheng Luan, Guangfeng Qiang, Baoxiang Cao, Kun Luo, Xianhong Meng, Baolong Chen, Jie Kong

**Affiliations:** 1 Key Laboratory for Sustainable Utilization of Marine Fisheries Resources, Ministry of Agriculture, Yellow Sea Fisheries Research Institute, Chinese Academy of Fishery Sciences, Nanjing Road 106, Qingdao, 266071 China; 2 Function Laboratory for Marine Fisheries Science and Food Production Processes, Pilot National Laboratory for Marine Science and Technology (Qingdao), No. 1 Wenhai Road, Aoshanwei Town, Jimo, Qingdao, China

## Abstract

**Background:**

Competition is a common social interaction among shrimp and depending on its intensity, it can affect heritable variation and response to selection. Little is known about the variance of indirect genetic effects (IGE) under competitive and non-competitive conditions in shrimp. In this study, we used extended mixed linear models to estimate genetic parameters for the direct genetic effect (DGE) and IGE on body weight in *Litopenaeus vannamei* raised under ad libitum (AF, non-competitive environment) and restricted (RF, competitive environment) feeding regimes.

**Results:**

Estimates of heritabilities for body weight obtained with a traditional animal model (i.e. without accounting for IGE) were 0.11 ± 0.09 under AF and 0.25 ± 0.11 under RF. With extended animal models that accounted for IGE, the corresponding estimates for body weight were 0.07 ± 0.08 and 0.34 ± 0.11. Thus, heritabilities were higher under the RF regime than under the AF regime, regardless of whether IGE was accounted for or not. The log-likelihood ratio test revealed significant IGE under the RF regime. Although estimates of indirect genetic variance were low (0.0023 ± 0.0013 for AF and 0.0028 ± 0.0012 for RF), they contributed substantially to the total heritable variance: 66.8% for AF and 692.2% for RF. The total heritable variance was smaller under the RF regime (0.7 ± 1.3) than under the AF regime (5.8 ± 2.6) because of the high contribution of the negative covariance between DGE and IGE (− 7.03). Estimates of the correlation between DGE and IGE were 0.32 ± 0.47 under AF and − 0.93 ± 0.15 under RF, those of DGE and IGE for body weight between both regimes were 0.94 ± 0.07 and 0.67 ± 0.20, respectively, and those of IGE for body weight with DGE for survival were − 0.12 ± 0.22 under AF and − 0.58 ± 0.20 under RF.

**Conclusions:**

These results indicate that strong competitive interactions occurred under the RF regime in *L. vannamei*. Significant reranking and variation in IGE of individuals were observed between the two feeding regimes. Strong competitive interactions reduced the total heritable variation for body weight when food was restricted. These results indicate that the extent of competition among *L. vannamei* depends on the feeding regime applied and that this competition affects the genetic basis of body weight.

## Background

Cannibalistic and aggressive behaviors are often observed in crustaceans [1, 2] and fish at high stocking density and low feeding frequency [3]. Such social interactions can affect growth, survival and welfare of the members of a population. For example, Metcalfe [4] reported that, in rainbow trout (*Salmo gairdneri*), dominant individuals access more food and grow faster than subordinates. In teleost fish, up to 90% of the mortality is due to cannibalism [5]. Inter-individual competition and dominance hierarchy have been reported to increase the coefficient of variation of body weight in Nile tilapia *(Oreochromis niloticus)* and Arctic charr (*Salvelinus alpinus* L.) [6, 7]. High variability of body size can affect growth, survival, and well-being of animals, and poses a serious obstacle to production efficiency in aquaculture. Management measures, such as size-grading, are necessary to minimize competition and increase uniformity, although it is labor intensive and stressful to animals [8, 9]. Social interactions between individuals can have a genetic component, which are known as indirect genetic effects (IGE), and occur when the genotype of an individual affects the phenotypic values of the individuals it interacts with [10].

For a family-based selective breeding program in aquaculture, candidates are usually tagged and tested in one or several communal rearing environments. Although observed social interactions may suggest the presence of IGE, these genetic effects cannot be estimated with a traditional animal model because of confounding with the direct genetic effect (DGE) and the lack of an effective experimental design. To estimate IGE, individuals need to be split into a large number of groups, which makes the data structure based on a traditional communal rearing environment unsuitable to separate DGE from IGE. However, ignoring IGE in selective breeding can lead to increased competition and a negative selection response [10, 11]. Experimental designs with multiple groups, with each group consisting of members of two or three families [12–14], and an extended animal model [11] have been proposed to estimate IGE. For example, variance estimates of IGE on fin damage traits in Atlantic cod (*Gadus morhua*) [15] were obtained by using a design that comprised 100 small groups (each group consisting of 21 fish from three families of seven fish). In Nile tilapia, an optimal design of multiple blocks of 11 families was used to estimate IGE on harvest weight [6, 14]. Significant IGE on body weight and a negative genetic correlation between DGE and IGE were obtained, which imply that heritable competitive interactions affect this trait in Nile tilapia. To increase response to selection for socially-affected traits, group selection and multilevel selection on IGE have been proposed [16–21].

More than 60 selection experiments have been carried out for increased growth in aquaculture species. Estimates of genetic gain for body weight obtained in these experiments ranged from 2.3 to 42% per generation, with an overall average of 12.7% [22]. Empirical evidence suggests that fast growing fish may be more aggressive and competitive under conditions of limited resources [23, 24]. In contrast, some geneticists argue that artificial selection for rapid growth may indirectly retain unaggressive fish when food is available in excess [25–27]. Whether aggression and competition increase or decrease because of artificial selection for growth may depend on whether food is available in restrictive or excessive amounts. However, there are few reports on the effect of selection for rapid growth on aggressive and competitive behaviors.

The Pacific white shrimp, *Litopenaeus vannamei*, is an important farmed shrimp worldwide, with an annual production of 4.46 million metric tons, accounting for 80% of the total penaeid shrimp production [28]. A family-based selective breeding program has resulted in significant genetic gains for growth and disease resistance in *L. vannamei* [29, 30]. In *L. vannamei*, the variance of IGE on adult body weight was found to be large at low stocking density and under an ad libitum feed regime [31]. There was no significant evidence for competitive interactions under these environmental conditions [31]. Selection response for IGE on the target trait depends on the competitive intensity of the rearing environments [8]. A trade-off between DGE and IGE occurs and the heritable variation gradually decreases when a strong negative correlation exists between DGE and IGE. Therefore, for a selective breeding program that aims at improving the effects of social interactions, it is important to test the genetic parameters for IGE on the target trait under different competitive environments. Few studies have investigated genetic parameters for IGE under an environment of limited resources and genotype-by-environment interactions for IGE in competitive versus non-competitive environments.

Thus, the main objective of this study was to quantify DGE and IGE for body weight under ad libitum (AF) and restricted (RF) feeding regimes in *L. vannamei.* Moreover, the type and intensity of social interactions among individuals under two feeding regimes were evaluated by estimating correlations between DGE and IGE for body weight. To investigate the consequences of selection for rapid growth on aggressive and competitive behaviors, genotype-by-environment interaction for IGE was evaluated by estimating the correlation of IGE for body weight between two feeding regimes.

## Methods

### Shrimp population

The social interaction experiment started in 2015 at Hebei Xinhai aquaculture Technology Ltd (longitude 110.952874, latitude 19.937534) in Huanghua City, Hebei Province, China. The experimental shrimp belonged to generation G_3_ of a selection line that was established in 2012. Eight improved batches from different companies in the United States and Singapore were introduced as founder broodstocks. Each shrimp was tagged on one ocular peduncle with a numbered ring. The shrimp were checked for different viruses and bacterial strains and those that were pathogen-free were reserved as broodstocks. The healthy broodstocks with mature gonads were chosen after 1 month of temporary rearing. The base population (generation G_0_) of eight strains was established by an incomplete diallel cross design. Each generation, full-sib and half-sib families were produced using a nested mating design, in which two dams (sires) were mated to the same sire (dam) by artificial insemination. In total, 207 full-sib families (40 paternal half-sib families; 59 maternal half-sib families) from 187 sires and 174 dams were produced in 2012. These families were tagged with a visible implant elastomer (VIE) and communally reared in two 100-m^2^ tanks for the grow-out test.

Candidates in generation G_0_ were ranked using a selection index that included the standardized individual estimated breeding values (EBV) for body weight and survival, with weights of 70 and 30%, respectively. The mating schemes for the selection and control populations in generation G_1_ were as follows: the selection population was generated by mating selected males and females with a high selection index, while maintaining a relatively large effective population size and controlling inbreeding (e.g., avoiding full-sib, half-sib and cousin mating). The control population was composed of 10 to 20 full-sib families produced by single-pair mating of 20 to 30 breeding candidates of generation G_0_. One hundred and five families (10 paternal half-sib families; 26 maternal half-sib families) were produced in generation G_1_ from 100 sires and 91 dams. The same scheme was used to generate generations G_2_ and G_3_. Eighty-seven families (24 paternal half-sib families; 30 maternal half-sib families; 33 full-sib families not nested in any of the half-sib family groups) from 65 males and 68 females of generation G_2_ were selected for the analysis of IGE in generation G_3_ (Table 1).

**Table 1:** Data structure and summary statistics for body weight (g) at the end of test under two feeding regimes in generation G_3_ of *Litopenaeus vannamei*

Test program^a^	Number of groups	Sex	N1^b^	N2	Mean	Min	Max	SD	CV	Survival rate (%)
*Ad libitum* feeding	80	All	3360	2641	16.82	4.40	31.50	3.67	21.82	78.60
		Male	–	1176	16.75	4.40	27.40	3.50	20.89	–
		Female	–	1435	16.87	4.50	31.50	3.80	22.56	–
Restricted feeding	80	All	3360	2640	14.89	4.60	28.90	3.91	26.26	78.57
		Male	–	1253	14.72	4.60	27.20	3.62	24.59	–
		Female	–	1387	15.04	4.80	28.90	4.14	27.53	–

N1, number of individuals at stocking

N2, number of individuals at harvest

Min, minimum body weight (g)

Max, maximum body weight (g)

SD, standard deviations of body weight (g)

CV, variation coefficient of body weight (%)

^a^Numbers of sires, dams, paternal and maternal half-sib families were equal to 65, 68, 24 and 30, respectively, but the number of half-sib families indicated is less than 54 because seven full-sib families were identical between the paternal and maternal groups

^b^Genders of individuals at stocking were not identified

### Experimental design

DGE and IGE on body weight were tested under the AF and RF feeding regimes at the group level using the 3FAM design, which is an optimum design of three families per group, where each family is tested repeatedly in three groups that each include two other families [15, 18]. The shrimp were fed four times a day with a commercial feed containing $\ge$ 41% crude protein, $\ge$ 6% crude fat, $\le$ 5% crude fiber, and $<$ 16% crude ash. The diameter of the feed pellets ranged from 0.5 to 1.6 mm according to shrimp size. Under the AF regime, feed intake per day represented 5 to 7% of body weight at the cage level. Total feed intake per day under the RF regime was equal to 50% of the feed intake under the AF regime. For each feeding regime, 42 shrimp per family (3360 individuals for 80 families of the selection line) were tagged with VIE and equally divided into three groups when the average body weight reached ~ 6.5 g. Next, one of the three groups from each family was randomly assigned to one of 80 net cages (70 × 70 × 100 cm^3^) in a rectangle concrete tank (100 m^2^), with restrictions to control coancestry coefficients (e.g., avoiding assigning half-sib and cousin families to the same cage) and to maintain a low coefficient of variation of body weight among the three families in a cage. Each cage had 42 shrimp from three families, which resulted in an average shrimp density of about 171 individuals/m^3^ (depth of the water of 0.5 m) at the beginning of the 71-day experiment. This stocking density is not high for most intensive culture models of *L. vannamei* [32]. The average density was about 134 individuals/m^3^ at the end of the experiment. The water exchange rate for each tank was about 20 to 30% every 3 days. Mortality rates under the AF and RF regimes were 21.40 and 21.43%, respectively. In total, 6720 shrimp were assigned to 160 cages within 1 day, and 5281 shrimp were harvested over 1 days after the 71-day growth-test (Table 1).

### Statistical analyses

Genetic analysis of body weight was performed using ASReml 4.1 [33] using an animal model with or without IGE. The traditional single-trait animal model without IGE for body weight was:

1 $$y_{{BW_{iklm} }} = \mu + Sex_{l} + b_{1} TBW_{m} (Sex_{l} ) + a_{{d_{i} }} + c_{m} + t_{k} + e_{iklm},$$

while the extended animal model with DGE and IGE was:

2 $$y_{{BW_{ijklm} }} = \mu + Sex_{l} + b_{1} TBW_{m} (Sex_{l} ) + a_{{d_{i} }} + \mathop \sum \limits_{i \ne j}^{n - 1} a_{{s_{j} }} + c_{m} + t_{k} + e_{ijklm} ,$$

where $y_{{BW_{iklm} }}$ or $y_{{BW_{ijklm} }}$ is the observed body weight of the $i$-th shrimp; $\mu$ is the overall mean; $Sex_{l}$ is the fixed effect of the $l$-th gender; $TBW_{m} (Sex_{l} )$ is a linear covariate of stocking body weight nested within the $l$-th gender, $b_{1}$ is a regression coefficient; $a_{{d_{i} }}$ is the DGE of the $i$-th shrimp, with vector ${\mathbf{a}}_{\varvec{d}} \sim \left( {0,{\mathbf{A}}\sigma_{{a_{d} }}^{2} } \right)$, where ${\mathbf{A}}$ is the additive genetic relationship matrix among all shrimp, including 5856 individuals in generations G_0_ to G_3_, and $\sigma_{{a_{d} }}^{2}$ is the variance of DGE; $\mathop \sum \nolimits_{i \ne j}^{n - 1} a_{{s_{j} }}$ is the sum of the IGE of $n - 1$ associates ($n$ = 42) for the $i$-th focal individual in one net cage, with vector ${\mathbf{a}}_{{\mathbf{s}}} \sim \left( {0,{\mathbf{A}}\sigma_{{a_{s} }}^{2} } \right)$, where $\sigma_{{a_{s} }}^{2}$ is the variance of IGE; $c_{m}$ is the random effect common to the $m$^*th*^ full-sib family, with vector ${\mathbf{c}}\sim \left( {0, {\mathbf{I}}\sigma_{c}^{2} } \right)$, which includes the tank effect resulting from separate rearing of the full-sib family before stocking and one quarter of the non-additive (dominance) genetic effect common to full-sibs, where ${\mathbf{I}}$ is the identity matrix, and $\sigma_{c}^{2}$ is the variance of common environmental effect; $t_{k}$ is the random effect of the $k$-th test net cage, with vector ${\mathbf{t}}\sim (0, {\mathbf{I}}\sigma_{t}^{2}$), $\sigma_{t}^{2}$ is the variance of the test net cage effect; and $e_{iklm}$ or $e_{ijklm}$ is the random residual error of the $i$-th individual, with vector ${\mathbf{e}}\sim \left( {0, {\mathbf{I}}\sigma_{e}^{2} } \right)$, where $\sigma_{e}^{2}$ is the residual variance. The likelihood ratio test (LRT) was used to compare animal models with or without IGE for each feeding regime based on LRT = 2 × (lnL1–lnL0), where lnL1 is the log likelihood score of the animal model with IGE, and lnL0 is that of the animal model without IGE. The resulting value was evaluated using a Chi squared distribution with 2 degrees of freedom to determine if the IGE was significant.

The total breeding value (TBV) was defined as the total heritable effect of an individual on body weight in the studied population, and was calculated as [20]:

3 $${\text{TBV}}_{i} = a_{{d_{i} }} + \left( {n - 1} \right)a_{{s_{i} }} .$$

Therefore, the total heritable variance ($\sigma_{TBV}^{2}$), which represents the total heritable variance expressed on the scale of phenotypic variance among tested individuals, was calculated as:

4 $$\sigma_{TBV}^{2} = \sigma_{{a_{d} }}^{2} + 2\left( {n - 1} \right)\sigma_{{a_{ds} }} + \left( {n - 1} \right)^{2} \sigma_{{a_{s} }}^{2} ,$$

where $\sigma_{{a_{ds} }}$ is the covariance between the DGE and IGE.

The phenotypic variance ($\sigma_{p}^{2}$) was calculated as follows:

For Model 1:

5 $$\sigma_{p}^{2} = \sigma_{{a_{d} }}^{2} + \sigma_{c}^{2} + \sigma_{t}^{2} + \sigma_{e}^{2} ,$$

For Model 2:

6 $$\sigma_{p}^{2} = \sigma_{{a_{d} }}^{2} + \left( {n - 1} \right)\sigma_{{a_{s} }}^{2} + \sigma_{c}^{2} + \sigma_{t}^{2} + \sigma_{e}^{2} .$$

Narrow-sense heritability in classical quantitative genetics theory is calculated as $h^{2} = \sigma_{{a_{d} }}^{2} /\sigma_{p}^{2}$. By analogy, the total heritable variance relative to the phenotypic variance, is defined as $T^{2} = \sigma_{TBV}^{2}$/$\sigma_{p}^{2}$. Therefore, the contribution of IGE to the heritable variance is inferred by comparing $T^{2}$ with $h^{2}$.

The correlation between DGE and IGE is defined as $r_{{a_{{d_{BW} s_{BW} }} }} = \sigma_{{a_{ds} }} /\sqrt {\sigma_{{a_{d} }}^{2} \sigma_{{a_{s} }}^{2} }$. This correlation reflects the type and intensity of social interactions, with a positive value indicating cooperative interaction, and a negative value indicating competitive interaction [17]. This implies that the number of cooperative interactions in the population is larger when this correlation is closer to 1, and the number of competitive interactions is larger when this correlation is closer to − 1.

The genotype-by-environment interaction of DGE and IGE on body weight between the two feeding regimes was analyzed by estimating the genetic correlations of DGE and IGE for body weight between the AF and RF regimes using the following two-environmental analysis model:

7 $$y_{{BW_{ijklm} }} = \mu + FR_{q} + Sex_{l} \left( {FR_{q} } \right) + b_{1} TBW_{m} \left( {FR_{q} \cdot Sex_{l} } \right) + a_{{d_{i} }} \left( {FR_{q} } \right) + \mathop \sum \limits_{i \ne j}^{n - 1} a_{{s_{i} }} \left( {FR_{q} } \right) + c_{m} + t_{k} \left( {FR_{q} } \right) + e_{ijklm} \left( {FR_{q} } \right),$$

where some terms are the same as in Model 2; $FR_{q}$ is the fixed effect of the $q$-th feeding regime; the terms in brackets nests the term outside the brackets; the heterogeneous variance structure (CORGH) in ASReml was used for estimating variances (covariances) of DGE and IGE between the two feeding regimes. The covariance between DGE and IGE for each feeding regime and the covariance between DGE for the AF regime and IGE for the RF feeding regime were not estimated because of problems with convergence.

The correlation of DGE for the AF regime with IGE for the RF regime was estimated using Model 7 with an extended factor analytic variance structure (XFA). Because of convergence issues, IGE for the AF regime and the common environmental effects for full sibs ($c$) for the two regimes were not included. Therefore, in the XFA structure we did not consider the variance of IGE for the AF regime, the covariances between IGE for the AF regime and the three other genetic effects (DGE for both feeding regimes and IGE for the RF regime), and the common environmental variances for both feeding regimes.

Variance components for survival were estimated using the following animal threshold model:

8 $$y_{{SUR_{ik} }} = \left\{ {\begin{array}{*{20}c} {0, \eta_{ik} \le 0} \\ {1, \eta_{ik} > 0} \\ \end{array} } \right.; \eta_{ik} = \mu + a_{i} + t_{k} + e_{ik} ,$$

where $y_{{SUR_{ik} }}$ represents survival status (1 = alive, 0 = dead) of the $i$-th shrimp; $\eta_{ik}$ is the underlying liability of $y_{{SUR_{ik} }}$, assumed to follow a cumulative standard normal distribution; $\mu$ is the overall mean; $a_{i}$ is the additive genetic effect of the $i$-th shrimp, with vector ${\mathbf{a}}$ ~ (0, ${\mathbf{A}}\sigma_{a}^{2}$), $\sigma_{a}^{2}$ is the additive genetic variance; $t_{k}$ is the random effect of the $k$-th test net cage, with vector ${\mathbf{t}}\sim (0, {\mathbf{I}}\sigma_{t}^{2}$), $\sigma_{t}^{2}$ is the variance of the test net cage effect; and $e_{ik}$ is the random residual error of the $i$-th individual, with vector ${\mathbf{e}}\sim \left( {0, {\mathbf{I}}\sigma_{e}^{2} } \right)$, where $\sigma_{e}^{2}$ is the residual variance and is assumed to equal 1. Heritability for survival on the underlying liability scale for survival was defined as $h_{u}^{2}$ = $\sigma_{a}^{2}$/($\sigma_{a}^{2} + \sigma_{t}^{2} + \sigma_{e}^{2} )$ and then was transformed to the observed scale using $h_{p}^{2} = h_{u}^{2} \frac{{z^{2} }}{{p\left( {1 - p} \right)}}$ [34], where $p$ is the proportion of a given survival rate in the data, and $z$ is the height of the ordinate of the normal distribution corresponding to a truncation point applied to proportion $p$ of survival after the grow-out test.

A bivariate model was used to estimate the correlation between IGE for body weight and DGE for survival under the AF and RF regimes, using Model 2 for body weight and the threshold model of Model 8 for survival. The us() variance structure in ASReml was used to estimate the variance–covariance structure of DGE and IGE for body weight with DGE for survival. Due to missing records on the body weight of dead individuals and other convergence issues, under the AF regime, common environmental effects of full sibs ($c$) and the effects of test cages ($t$) were not estimated for body weight and the $c$ effect was not estimated for survival. Under the RF regime, the $c$ effect for survival was also not obtained because of convergence problems.

## Results

### Descriptive statistics

The average body weight at the end of the test was greater under the AF regime (16.8 g) than under the RF regime (14.9 g), whereas the standard deviation and coefficient of variation of body weight were lower under the AF regime (3.7 g and 21.8%) than under the RF regime (3.9 g, and 26.3%). The RF regime may have increased competition among individuals that shared the same environment and food. However, there was no difference in survival between the AF and RF regimes.

### Variance components and genetic parameters

Table 2 shows the estimates of variance components and genetic parameters for body weight using the model with or without IGE. Estimates of narrow-sense heritability for body weight were higher under the RF regime than under the AF regime regardless of whether IGE were included in the model or not. Based on the LRT, IGE should be included in the model under the RF regime ($\chi^{2}$ = 14.22*, P *< 0.001). The small variances for IGE of body weight, 0.0023 ± 0.0013 and 0.0028 ± 0.0012 under the AF and RF regimes, respectively, contributed substantially to the total heritable variance. The contributions of the variance of IGE over the total heritable variance ($\frac{{\left( {n - 1} \right)^{2} \sigma_{{a_{s} }}^{2} }}{{\sigma_{TBV}^{2} }} \times 100\%$) were equal to 66.78 and 692.18% under the AF and RF regimes, respectively. The total heritable variance under the AF regime was seven times larger than the direct genetic variance but the total heritable variance under the RF regime was much smaller than the ordinary direct genetic variance due to the high contribution of the negative covariance between DGE and IGE ($\frac{{2\left( {n - 1} \right)\sigma_{{a_{ds} }} }}{{\sigma_{TBV}^{2} }} \times 100\%$ = − 1049.12%). Consequently, the $T^{2}$ estimated with the model with IGE was much lower than the $h^{2}$ estimated with the classic model without IGE.

**Table 2:** Estimates of genetic parameters for body weight (g) based on models without or with indirect genetic effects (IGE) under two feeding regimes in *Litopenaeus vannamei*

Parameter	Model without IGE		Model with IGE	
	*Ad libitum* feeding	Restricted feeding	*Ad libitum* feeding	Restricted feeding
Log likelihood	− 4301.81	− 4027.37	− 4299.40	− 4020.26
$\sigma_{{a_{d} }}^{2}$	1.15 ± 0.97	2.26 ± 1.07	0.75 ± 0.86	3.17 ± 1.16
$\sigma_{{a_{s} }}^{2}$	–	–	0.0023 ± 0.0013	0.0028 ± 0.0012
$\sigma_{{a_{ds} }}$	–	–	0.013 ± 0.017	-0.087 ± 0.028
$\sigma_{c}^{2}$	0.76 ± 0.43	0.36 ± 0.38	0.86 ± 0.42	0.38 ± 0.35
$\sigma_{t}^{2}$	0.37 ± 0.12	0.46 ± 0.13	0.025 ± 0.13	0.31 ± 0.13
$\sigma_{TBV}^{2}$	–	–	5.79 ± 2.57	0.68 ± 1.28
$\sigma_{e}^{2}$	8.23 ± 0.54	5.96 ± 0.57	8.46 ± 0.50	5.37 ± 0.63
$\sigma_{p}^{2}$	10.53 ± 0.38	9.04 ± 0.39	10.20 ± 0.37	9.35 ± 0.49
$T^{2}$	–	–	0.57 ± 0.25	0.07 ± 0.14
$h^{2}$	0.11 ± 0.09	0.25 ± 0.11	0.07 ± 0.08	0.34 ± 0.11
$c^{2}$	0.07 ± 0.04	0.04 ± 0.04	0.09 ± 0.04	0.04 ± 0.04
$r_{{a_{{d_{BW} s_{BW} }} }}$	–	–	0.32 ± 0.47	− 0.93 ± 0.15
Two-environmental analysis model with heterogeneous variance structure for body weight				
$r_{{g_{{d_{BW} d_{BW} }} }}$	0.94 ± 0.06		0.94 ± 0.07	
$r_{{g_{{s_{BW} s_{BW} }} }}$	–		0.67 ± 0.20	
Two-environmental analysis model with an extended factor analytic variance structure for body weight^a^				
$r_{{g_{{d_{BW} s_{BW} }} }}$	–		− 0.81 ± 0.12	
Bivariate model for body weight and survival				
$r_{{a_{{s_{BW} d_{SUR} }} }}$	–		− 0.12 ± 0.22	− 0.58 ± 0.20
$r_{{a_{{d_{BW} d_{SUR} }} }}$	–		0.71 ± 0.10	0.71 ± 0.12

^a^Correlation for body weight between IGE for the ad libitum feeding regime (AF) and DGE for the restricted feeding regime (RF) was not estimated because the model with IGE of body weight for the AF regime did not converge

IGE, indirect genetic effects

$\sigma_{{a_{d} }}^{2}$, direct genetic variance

$\sigma_{{a_{s} }}^{2}$, indirect genetic variance

$\sigma_{{a_{ds} }}$, direct–indirect genetic covariance

$\sigma_{c}^{2}$, common environmental variance

$\sigma_{t}^{2}$, variance of the test tank effect

$\sigma_{TBV}^{2}$, variance of the total breeding value

$$\sigma_{e}^{2}$$, residual variance

$$\sigma_{p}^{2}$$, phenotypic variance

$$T^{2}$$, total heritability

$h^{2}$, narrow sense heritability

$c^{2}$, common environmental coefficient

$r_{{a_{{d_{BW} s_{BW} }} }}$, correlation between DGE and IGE of body weight

$r_{{a_{{s_{BW} d_{SUR} }} }}$, correlation between IGE of body weight and DGE of survival

$r_{{a_{{d_{BW} d_{SUR} }} }}$, correlation between DGE of body weight and DGE of survival

$r_{{g_{{d_{BW} d_{BW} }} }}$, correlation on DGE of body weight between the AF and RF regimes

$r_{{g_{{s_{BW} s_{BW} }} }}$, correlation on IGE of body weight between the AF and RF regimes

$r_{{g_{{d_{BW} s_{BW} }} }}$, correlation in body weight between DGE for the AF regime and IGE for the RF regime

The common environmental coefficient estimated by using different models ranged from 0.04 ± 0.04 to 0.09 ± 0.04 for the AF and RF regimes (Table 2). The variances of the test net cage effect for the AF and RF regimes accounted for 0.25 to 5.09% of the phenotypic variance, and were small because the net cages used for either regime were in the same water environment. Inclusion of IGE reduced the variance of the net cage effect (Table 2), which indicates that the net cage effect was caused partly by social interactions among individuals and not entirely by physical differences between cages.

The negative and significant estimate of the correlation between DGE and IGE under the RF regime (− 0.93 ± 0.15; z-score = − 6.2, *P* < 0.01) indicates that strong competitive interactions occurred when the amount of food provided was limited. In contrast, the positive correlation between the DGE and IGE under the AF regime (0.32 ± 0.47) implies that there was no evidence of competitive interactions when food was available in excess.

Estimates of genetic correlations to quantify genotype-by-environment interactions for DGE and IGE on body weight between the two feeding regimes are in Table 2. The estimate of the correlation between the AF and RF regimes was positive and high for DGE (0.94 ± 0.07) and medium positive for IGE (0.67 ± 0.20; z-score = − 1.65, *P* < 0.05). This indicates that a re-ranking for IGE of the families occurred between the two competitive levels. The high negative correlation estimate of DGE under the AF regime with IGE for the RF regime (− 0.81 ± 0.12) implies that retained individuals characterized by a rapid growth under the AF regime would show strong competition when tested under the RF regime.

The back-transformed heritabilities of survival under the AF and RF regimes were 0.08 ± 0.02 and 0.07 ± 0.02, respectively (Table 3). The estimates of the correlation of IGE for body weight with DGE for survival were − 0.12 ± 0.22 under the AF regime and − 0.58 ± 0.20 under the RF regime (Table 2). This suggests that shrimp that are genetically more competitive may survive better and suppress the growth of group mates.

**Table 3:** Genetic parameters estimated for survival using a threshold model under two feeding regimes in *Litopenaeus vannamei*

Feeding regime	Parameter					
	$\sigma_{a}^{2}$	$\sigma_{t}^{2}$	$\sigma_{e}^{2}$	$\sigma_{p}^{2}$	$h_{u}^{2}$	$h_{p}^{2}$
*Ad libitum* feeding	0.18 ± 0.04	0.03 ± 0.01	1	1.20 ± 0.04	0.15 ± 0.03	0.08 ± 0.02
Restricted feeding	0.18 ± 0.04	0.09 ± 0.02	1	1.26 ± 0.05	0.14 ± 0.03	0.07 ± 0.02

$\sigma_{a}^{2}$, additive genetic variance

$\sigma_{t}^{2}$, variance of the test tank effect

$$\sigma_{e}^{2}$$, residual variance

$$\sigma_{p}^{2}$$, phenotypic variance

$h_{u}^{2}$, heritability on underlying liability scale

$h_{p}^{2}$, back-transformed heritability on observed scale

## Discussion

### Overall findings

This study reports the first large-scale test for IGE under different competitive environments in aquaculture. A strong re-ranking of the genetic competitive abilities of families was detected between the two feeding regimes. Individuals that were genetically highly competitive showed better survival under the RF regime. The total heritable variance of body weight was greatly reduced because of the strong competitive interactions that occurred among individuals under the RF regime. The results indicate that an increase or a decrease in competition during artificial selection for growth depends on resource availability. Moreover, the results indicate a cryptic genetic variation for body weight when feed is limited.

### Differences in body weight between the AF and RF regimes

Differences in body weight between the AF and RF feeding regimes were relatively small although the RF regime represented 50% less feed intake compared to the AF regime (Table 1). However, the net cages were not cleaned during the study and a lot of algae grew on the surface of the net cages, which the shrimp under the RF regime ate. Moreover, the feed was provided in excess to ensure that the shrimp in all net cages were fed ad libitum under the AF regime. Therefore, the real feed intake per shrimp under the RF regime was probably more than 50% of feed intake of the shrimp fed ad libitum. In addition, feed efficiency of individuals under the RF regime could have been higher than that under the AF regime. In an eight-generation selected population of the black tiger shrimp, the feed conversion ratios (FCR) of shrimp fed 75% of two diets were lower than FCR of shrimp fed 100% (2.32 vs 2.44; 2.92 vs 3.14) [35]. In addition, Abdel-Tawwab et al. [36] reported that Nile tilapia individuals that were fasted for 1 to 3 weeks had lower weekly FCR than control individuals after refeeding for up to 13 weeks. Lower FCR were also recorded in a carp-prawn polyculture system under two restricted feeding regimes compared to a regular feeding regime [37].

### Bias in estimates of genetic parameters

In this study, estimates of heritability for body weight under the AF regime (0.07 ± 0.08 and 0.11 ± 0.09) were lower than previously reported estimates in *L. vannamei* (0.17 ± 0.04 to 0.45 ± 0.09) [38–41], which could be due to the 3FAM design. One family was tested only against six other families in three different cages to estimate IGE in the 3FAM design. However, an optimal design for estimating the additive genetic variance requires that all families are communally reared in one (or a few) large tank(s). Thus, our estimates of heritability may be biased. The low heritability estimates for body weight may also be due, at least in part, to the small number of full-sib families. Only 80 full-sib families were used because of restricted access to facilities, compared to other reports that included up to 448 full-sib families [38]. Thus, the additive genetic variance estimated by using these families may only account for part of the total genetic variance from the whole breeding nucleus population.

Estimates of correlations between IGE of body weight and DGE of survival may be biased because the $c$ effect for survival was not included in the model due to convergence problems. Heritabilities of survival were also overestimated due to the confounding between additive genetic and common environmental effects. The $c$ effect for survival is difficult to partition because of the binomial nature of survival data, especially when genetic ties between families are weak. A larger number of half-sib families with a deep pedigree and test groups would help to partition the $c$ effect for survival.

The estimate of the correlation of DGE on body weight under the AF regime with IGE on body weight under the RF regime ($r_{{g_{{d_{BW} s_{BW} }} }}$ = − 0.81 ± 0.12) might also be biased because the analysis model includes neither IGE under the AF regime, nor the $c$ effect for both regimes due to convergence problems. However, common environmental variances (0.36 to 0.86) were relatively small compared to the phenotypic variances (9.04 to 10.53) and the contribution of IGE variance (3.87 to 4.71). IGE were not significant under the AF regime ($\chi^{2}$ = 4.82, *P *> 0.05). The estimate of the correlation between DGE and IGE for body weight under the AF regime was also small (0.32 ± 0.47). Moreover, the correlation of DGE between both regimes (0.94 ± 0.07) and the correlation between DGE and IGE under the RF regime (− 0.93 ± 0.15) were all high. Therefore, the bias in $r_{{g_{{d_{BW} s_{BW} }} }}$ could be very small by using the above model.

Standard errors of the genetic parameter estimates for IGE were high and influenced by the number of test groups. For a dataset of 493 test groups in Nile tilapia *O. niloticus*, small standard errors of the estimate of the variance of IGE for body weight were obtained [6]. A larger number of groups (250–500) and a large group size (60) are required for accurate estimation of genetic parameters for IGE when the heritability of social effects is low [16, 17]. The number of half-sib families may also affect the precision of the genetic parameters. Variances of DGE and IGE could not be estimated in [6] when the number of half-sib families was small, because the common environmental effect was confounded with DGE. Rearing density may also affect the standard errors of estimates of genetic parameters of IGE. When the rearing density is low, social interactions among shrimp are rare and difficult to detect. In an analysis of IGE for adult body weight of *L. vannamei* at low harvest rearing density (14 individuals/m^3^) [31], the standard error (0.0031 ± 0.039) obtained for the estimate of the IGE-DGE covariance was large. In the current study, with a relatively high harvest rearing density (134 individuals/m^3^), relatively small standard errors were obtained for estimates of the IGE-DGE covariance (− 0.087 ± 0.028 to 0.013 ± 0.017). Nevertheless, a larger standard error for the estimate of the correlation between DGE and IGE under the AF regime (0.32 ± 0.47) than under the RF regime (− 0.93 ± 0.15) implies that competitive interactions were less frequent under the AF regime.

### Strong competitive interactions and total heritable variance

Very strong competitive interactions were observed under the RF regime, based on the estimate of the genetic correlation between DGE and IGE of − 0.93 ± 0.15. The IGE of an individual for body weight rapidly spreads to all its mates, and its full impact is reflected in the TBV. The much smaller variance of TBV of body weight under the RF regime than under the AF regime (0.68 vs 5.79) implies that the total heritable variance was greatly reduced by the strong competitive interactions under the limited feeding condition. Although the difference between the estimates of the variance of IGE for the RF and AF regimes was small (0.0028 ± 0.0012 vs 0.0023 ± 0.0013), the difference between the estimates of the covariance between DGE and IGE for the RF and AF regimes was large (− 0.087 ± 0.028 vs 0.013 ± 0.017). The large negative covariance decreased the total heritable variance under the RF regime by 91% ($\frac{{2\left( {n - 1} \right)\sigma_{{a_{ds} }} }}{{\sigma_{{a_{d} }}^{2} + (n - 1)^{2} \sigma_{{a_{s} }}^{2} }} \times 100\%$). Such a negative covariance could gradually reduce the heritable variance and severely reduce response to selection over generations. For blue gum (*Eucalyptus globulus*), Silva et al. [42] reported that IGE also reduced the total heritable variance for growth by 92% because of a negative covariance between DGE and IGE. For Nile tilapia, Khaw et al. [6] showed that the negative covariance between DGE and IGE completely eliminated the contribution of IGE variance to the total heritable variance ($\frac{{2\left( {n - 1} \right)\sigma_{{a_{ds} }} }}{{\sigma_{TBV}^{2} }} \times 100\%$ = − 55% vs $\frac{{\left( {n - 1} \right)^{2} \sigma_{{a_{s} }}^{2} }}{{\sigma_{TBV}^{2} }} \times 100\%$ = 48%). Moreover, Ellen et al. [43] reported that the accuracy of individual selection in Nile tilapia could be reduced by 22% due to a negative covariance between DGE and IGE as this covariance determines the impact of IGE on the total heritable variance and therefore potential response to selection. Actually, adverse interactions among individuals may lead to positive or negative covariance between DGE and IGE depending on the traits targeted. A positive covariance for aggressive behavior or disease susceptibility may occur under adverse interactions. For example, in mink *Neovison vison*, a positive genetic correlation (0.90 ± 0.15) and a large covariance between DGE and IGE (45% of the total genetic variance) were obtained for total bite mark score [44], and in *E. globulus*, the covariance between DGE and IGE increased the heritable variance by 71% for Mycosphaerella leaf disease [42].

### Artificial selection and competition

When designing breeding schemes, the consequences of artificial selection for growth or other economic traits on social interactions should be evaluated. Excessively aggressive individuals spend much time and energy chasing competitors on unnecessary attempts to monopolize the food supply, thereby allowing less aggressive individuals to acquire a growth advantage when food is available in excess [25]. In our study, the positive estimate of the genetic correlation between DGE and IGE on body weight under the AF regime supports this theoretical hypothesis. Excessively aggressive individuals may have been culled through the highly intense selection applied during the past 30 years of breeding in *L. vannamei* [45]. If aggressive individuals were present in the tested population, the correlation between DGE and IGE on body weight would have been zero or even negative. The observed positive correlation implies, to a certain extent, that there were schooling behaviors in the tested population. In Medaka, the overall level of agonistic interactions among group members was shown to decrease in an environment with excess food after performing two generations of selection for rapid growth [24]. In Atlantic salmon, no significant inter-strain competitions between farmed individuals of generations 9 to 10 and wild individuals were observed under the standard feeding regime, based on their relative growth when reared together or separately [46].

Artificial selection for growth increases the level of competitive interactions when the amount of food is limited. In our study, the negative and strong correlation between DGE and IGE on body weight under the RF regime implies that individuals with genetics for rapid growth also possess strong competitive abilities. This suggests that selection may increase competitive behaviors in *L. vannamei* under conditions with limited resources. In the literature, comparisons between domestic and wild stocks have shown large differences in growth or agonistic behaviors in coho salmon [23], brook trout [47], and common carp *Cyprinus carpio* [48]. In Japanese quail, a negative response to selection for weight at 6 weeks (− 0.10 ± 0.25 g/hatch) and a positive correlated response in mortality (0.32 ± 0.15 deaths/hatch) indicated that aggressive and cannibalistic behaviors increased over 23 cycles of selection when only DGE was used as the selection criteria under conditions with limited food and space resources [9].

The moderate correlation between IGE under the AF and RF regimes shows that reranking based on the competitive abilities of families occurred between the two feeding regimes. This implies that the condition with limited food caused and increased variations in competitive abilities of families. To our knowledge, this is the first time that a quantitative genetics analysis supports the assumption “increase or decrease in aggression and competition during selection depend on whether food is limited or available in excess” [26]. We show that the competitive abilities of families vary when food supply goes from adequate to limited.

In salmon, crawfish and giant freshwater prawn, aggressive behaviors typically occur when individuals fight for the right to monopolize food, to occupy space, and to acquire social dominance and hierarchy. However, these behaviors have rarely been observed in the improved selection population of *L. vannamei*. Large individuals may be more competitive than small individuals, given their higher growth rate and size advantage, termed “the size hierarchy effect”. The most competitive individuals can quickly search and find food because they have very good eyesight and strong locomotion systems [49]. Therefore, aggressive interactions in *L. vannamei* are not necessary for individuals to obtain food under limited conditions. The high correlation for body weight between DGE under the AF regime and IGE under the RF regime shows that the individuals that were selected might possess high levels of competitive ability, while no competitive interactions were detected under the AF regime. In contrast, a domesticated strain of rainbow trout did not express competitive ability compared to its wild strain under the limited food condition [50]. In fact, it is difficult to investigate the competitive ability of the selected or domesticated population because the competitive effect is confounded with the size hierarchy effect [46, 51].

### Cryptic genetic variation

In contrast to the small direct genetic variance (0.75 to 1.15) under the AF regime, a larger direct genetic variance (2.26 to 3.17) was observed under the RF regime. However, the phenotypic mean and variance for body weight were larger under the AF regime (mean BW = 16.8 g, $\sigma_{p}^{2}$ = 10.20 to 10.53) than under the RF regime (mean BW = 14.9 g, $\sigma_{p}^{2}$ = 9.04 to 9.35). These differences are the reverse of those observed at the direct genetic level. The greater genetic variation under the RF regime may be due to cryptic genetic variation (CGV) that is released under an atypical environment in which a population rarely lived during its selection history and generates heritable variation [52]. In our study, the selective breeding population was reared under ad libitum feeding conditions in the past generations. Therefore, CGV was released when the population was exposed to the environment with limited food. For example, in the threespine stickleback (*Gasterosteus aculeatus*), a small additive genetic variance (0.085) for body size was detected under high salinity, but it increased significantly (3.289) when an oceanic population was reared under low salinity [53]. Similarly, in farmed Atlantic salmon, the estimate of heritability of body weight was higher under a restricted hatchery treatment than under a control hatchery treatment (0.26 vs 0.16). In contrast, the estimate of the heritability of body weight in wild salmon increased significantly under the same two treatments (0.15 vs 0.51) [46]. CGV was also found for spermathecae number in dung flies [54], for plasma corticosterone level in gull chicks [55], and for traits that are associated with facultative carnivory in spadefoot [56] when abrupt ecological environment changes occurred. CGV provides a pool of standing genetic variation that is poised to facilitate genetic improvement when the rearing environment drastically changes.

## Conclusions

Our findings reveal strong competitive interactions among *L. vannamei* when reared under conditions with limited food. The competitive abilities of families differed significantly between the ad libitum and restricted feeding regimes. Strong competitive interactions reduced the total heritable variance for body weight when the amount of food was limited. Our results support the assumption that an increase or a decrease in aggression and competition during selection depends on whether the amount of available food is limited or excessive.

## Data Availability

The pedigree dataset used in this study cannot be made available because of commercial considerations. However, the phenotypic data are available from the authors upon request.
